# Primary ciliary dyskinesia in Japan: systematic review and meta-analysis

**DOI:** 10.1186/s12890-019-0897-4

**Published:** 2019-07-25

**Authors:** Atsushi Inaba, Masanori Furuhata, Kozo Morimoto, Mahbubur Rahman, Osamu Takahashi, Minako Hijikata, Michael R. Knowles, Naoto Keicho

**Affiliations:** 1 Department of Pathophysiology and Host Defense, the Research Institute of Tuberculosis, Japan Anti-tuberculosis Association, 3-1-24, Matsuyama, Kiyose, Tokyo 204-8533 Japan; 2 Department of Respiratory Medicine, Graduate School of Medicine, The University of Tokyo, 7-3-1, Hongo, Bunkyo-ku, Tokyo 113-8655 Japan; 3 Dapartment of Pathology, Graduate School of Medicine, The University of Tokyo, 7-3-1, Hongo, Bunkyo-ku, Tokyo 113-8655 Japan; 4 Department of Pathology, National Center for Global Health and Medicine, 1-21-1, Toyama, Shinjuku-ku, Tokyo 162-8655 Japan; 5 Fukujuji Hospital, Japan Anti-tuberculosis Association, Respiratory Disease Center, 3-1-24 Matsuyama, Kiyose, Tokyo 204-0022 Japan; 6 Center for Clinical Epidemiology, St. Luke’s International University Graduate School of Public Health, 3-6 Tsukiji, Chuo-ku, Tokyo 104-0045 Japan; 7 Department of Medicine and Marsico Lung Institute, University of North Carolina School of Medicine, Chapel Hill, North Carolina 27599 USA

**Keywords:** Systematic review, Meta-analysis, Primary ciliary dyskinesia, Immotile cilia syndrome, Kartagener syndrome, Electron microscopy

## Abstract

**Background:**

Primary ciliary dyskinesia (PCD) is a rare genetic disorder. Although the genetic tests and new diagnostic algorithms have recently been recommended, clinical signs and electron microscope (EM) findings have historically been the mainstays of diagnosis in Asia. To characterize PCD previously reported in Japan, we conducted a systematic review and meta-analysis.

**Methods:**

A search using MEDLINE, EMBASE, and Japana Centra Revuo Medicina (in Japanese) databases was carried out to identify articles reporting PCD, Kartagener syndrome, or immotile cilia syndrome in Japanese patients and published between 1985 and 2015.

**Results:**

After excluding duplication from 334 reports, we extracted 316 patients according to the criteria. Diagnosis was most frequently made in adulthood (148 patients [46.8%] ≥ 18 years old, 24 patients [7.6%] < 1 year old, 68 patients [21.5%] 1–17 years old and 76 patients [24.1%] lacking information). Of the 230 patients (72.8%) who received EM examination, there were patients with inner dynein arm (IDA) defects (*n* = 55; 23.9%), outer dynein arm (ODA) defects (14; 6.1%), both ODA and IDA defects (57; 24.8%), other structural abnormalities (25; 10.9%), no abnormalities (4; 1.7%), and no detailed conclusion or description (75; 32.6%).

**Conclusion:**

Delayed diagnosis of this congenital disease with high frequency of IDA defects and low frequency of ODA defects appear to be historical features of PCD reported in Japan, when EM was a main diagnostic tool. This review highlights problems experienced in this field, and provides basic information to establish a modernized PCD diagnosis and management system in the future.

**Electronic supplementary material:**

The online version of this article (10.1186/s12890-019-0897-4) contains supplementary material, which is available to authorized users.

## Background

Primary ciliary dyskinesia (PCD) is a rare genetic disorder with structural and/or functional abnormalities in cilia of various organs and flagella of sperm [1, 2]. PCD often presents as neonatal respiratory distress, hypoxia shortly after birth and situs anomaly, followed by chronic airway infection usually with infertility [3]. Although Kartagener syndrome was formerly known as a classical type of PCD with Kartagener triad; situs inversus, chronic sinusitis and bronchiectasis, the situs inversus appears to be observed in 40 to 50% of patients with PCD [4–6].

Currently combinations of several tests are proposed to make a diagnosis of PCD; nasal nitric oxide (NO) measurement, observation of ciliary structure under electron microscope (EM), genetic panel tests, ciliary-beat and -waveform analysis with high speed videomicroscopy (HSVM), and immunofluorescence (IF) test [3, 7]. For many patients in the world, however, the diagnostic tests are still challenging and not readily available. Thus, clinicians tend to diagnose them as having a non-specific chronic airway disease [7–9].

Most previous reports including causative genes of PCD have been published in western countries [2, 9–12]. Articles on PCD from Asian countries are mainly simple case reports and case series, although incidence of PCD in Asia may be higher than in western countries [13–15]. In this systematic review, we aimed to outline patients with PCD previously reported in Japan when the diagnosis was mainly based on EM findings (1985 to 2015); along with their clinical and laboratory findings, and diagnostic methods, and to compare the findings of this review with those reported from other countries, which may help facilitate establishment of a modernized PCD diagnosis and management system.

## Methods

### Search strategy

MEDLINE, EMBASE, and Japana Centra Revuo Medicina (*Ichushi* in Japanese) databases were searched electronically during November 15–28, 2016 supported by clinical librarians at St. Luke’s International University Library to identify the titles and abstracts reporting PCD in Japanese patients, published between 1985 and 2015 in Japanese and English. A combination of relevant medical subject heading terms along with keywords such as; “primary”, “cilia”, “ciliary”, “dyskinesia”, “disorders”, “Kartagener”, “syndrome”, “immotile”, “motility”, “Japan”, and “Japanese” were used (see details of the search strategy; Additional file 1). In addition, a manually searching method, ‘hand-search’ was applied by the authors (A. I. and M. F.) based on the reference lists of the relevant articles and web links suggested by the journal and conference abstract publishers. Finally, full text articles and conference abstracts of the identified citations were retrieved and reviewed to determine their eligibility for inclusion.

### Article selection

Two reviewers (A.I. and M.F.) independently read retrieved abstracts and titles, and initially assessed them according to the predefined inclusion and exclusion criteria. The inclusion criteria for the patients were: 1) diagnosis of PCD, Kartagener syndrome or immotile cilia syndrome was made, 2) the patients were Japanese in ethnicity, and 3) their age at the visit to clinicians and sex were reported. The exclusion criteria were: 1) definite diagnosis of PCD, Kartagener syndrome, or immotile cilia syndrome was not shown, 2) the patients were non-Japanese, and 3) their age at the visit to clinicians or sex were not described anywhere. Patients’ age, sex, author name, affiliations and other available information were used to identify the same patients in different articles. The full text of studies meeting these criteria was retrieved and screened to determine eligibility again. In addition, reference lists of the articles were reviewed and scrutinized for relevant papers. Discrepancies between the two reviewers were resolved by reconfirmation of the article contents.

### Data extraction

Using data abstraction forms, the information extracted by the two reviewers was as follows: study characteristics (the first author’s name and affiliation, study design, year of publication, paper’s title, and the number of patients reported), patients characteristics (sex, age at the diagnosis, age at onset of respiratory symptoms, main symptoms, family history, and medical history), laboratory findings (spirometry, genetic test, IF test, HSVM, saccharin test, nasal NO measurement, sputum culture test results, and EM analysis), macrolide therapy, other treatment, and outcome.

The age at the onset of symptoms was not reported clearly in many articles, using unspecified terms such as “childhood”, “around X years old” and “in elementary school”. To cope with this, we classified them into three groups; neonatal or infant period (< 1 year), childhood or adolescence (1–17 years) and adulthood (≥ 18 years). Following the original authors’ medical assessment described in their articles, we classified the patients’ outcomes into three groups; improvement, worsening, and death.

### Statistical analysis

We conducted quantitative synthesis for clinical presentation and made a summary of patient characteristics and laboratory findings. All statistical analyses were performed using R version 3.4.1. The summary included the frequency, mean or median of each clinical finding. We divided the patients into two subgroups by age at diagnosis (< 18 years and ≥ 18 years), and made comparisons by using chi-squared test, exact Wilcoxon rank sum test and Spearman’s rank correlation test, as appropriate. Patients < 18 years were not included in the statistical analysis of infertility.

We compared frequencies of EM findings in the present study with those in previously published reports by using chi-squared test [2, 10–12, 16]. Subgroup analysis on more than two categories of EM findings was also performed, using chi-squared test, Fisher’s exact test, or ANOVA, as appropriate. We could not calculate effect size and examine study quality, study heterogeneity across studies and publication bias due to summative nature of this systematic review.

Our protocol and search strategy are registered with and listed in PROSPERO (http://www.crd.york.ac.uk/PROSPERO [CRD42017076336]); this includes the search terms and keywords used. This study was in accordance with Preferred Reporting Items for Systematic Reviews and Meta-Analyses (PRISMA) guidelines [17]. The PRISMA checklist can be found in the supporting information (Additional file 2).

## Results

**Fig. 1:**
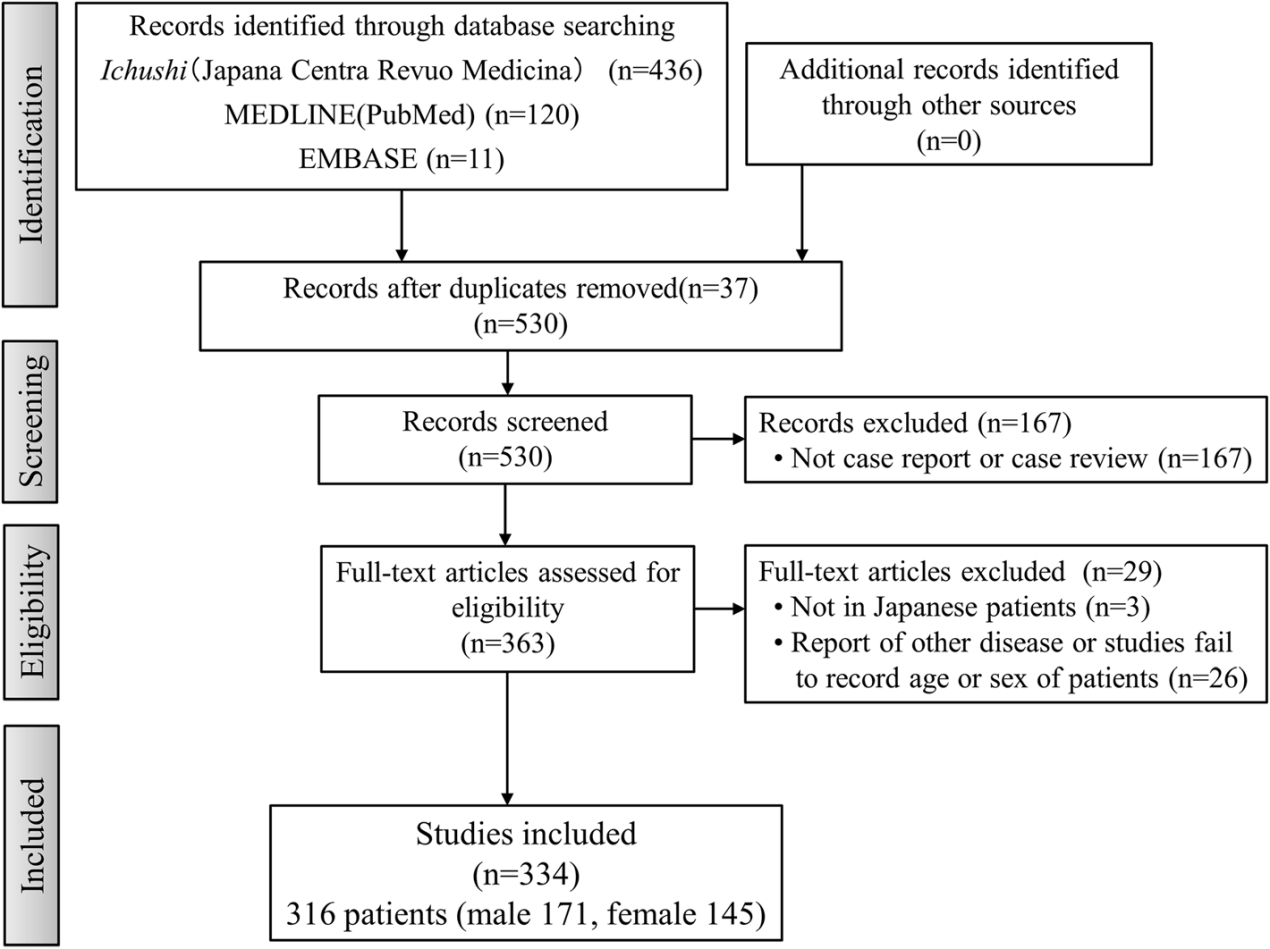
Study Flow Diagram

**Fig. 2:**
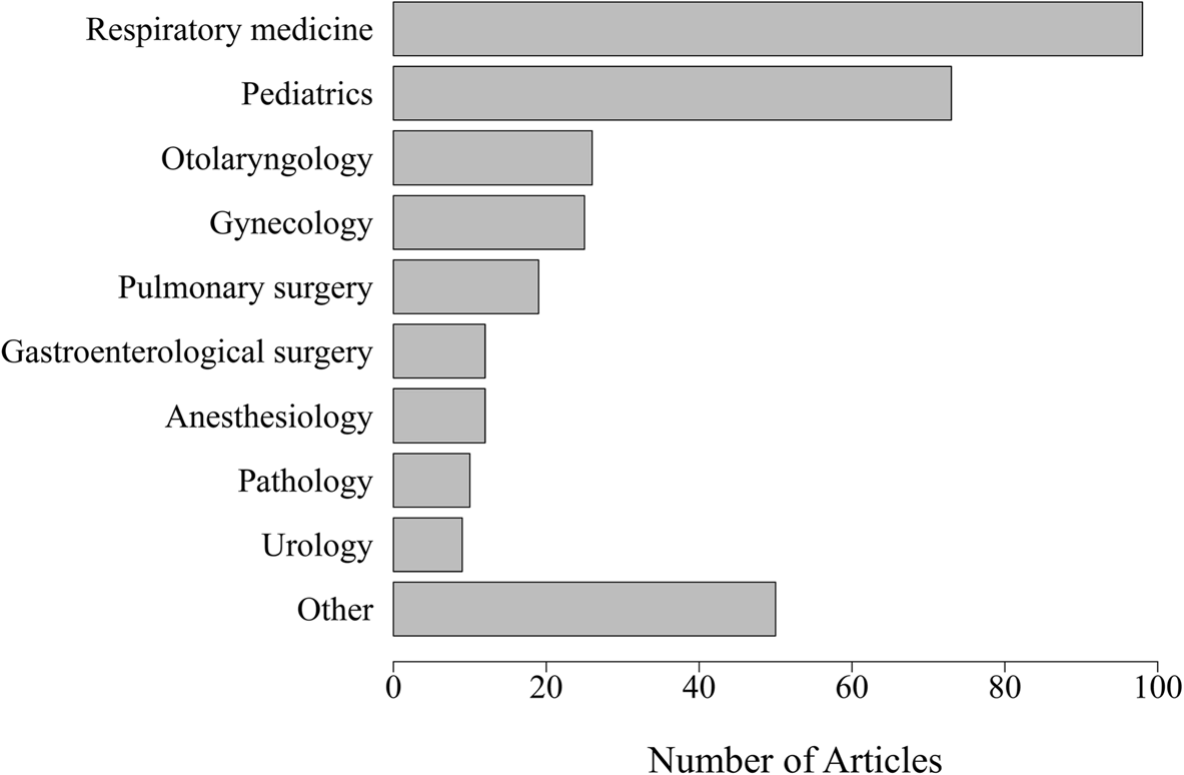
Authors’ affiliations (*n* = 334)

Our searches extracted 567 articles (MEDLINE 120, EMBASE 11, and Japana Centra Revuo Medicina 436), of which 334 articles met our eligibility criteria (Fig. 1). Analysis of the first authors’ affiliations illustrated that pulmonary physicians (98 articles, 29.3%) reported PCD most frequently in Japan (Fig. 2).

### Patients’ characteristics

Information of 316 patients (male; 171 patients [54.1%]) was collected from the 334 articles, omitting duplication, and their characteristics were summarized in Table 1. Slight male predominance was observed. Of these, 24 patients (7.6%) including 19 accompanied by situs inversus were diagnosed in their infancy; 68 patients (21.5%) diagnosed between 1 and 17 years; and 148 patients (46.8%) ≥ 18 years old. Of these, 60 patients (19.0%) had the onset of respiratory symptoms in neonatal period or infancy, 131 (41.5%) in childhood or adolescence and 28 (8.9%) in adulthood. Neonatal respiratory distress at term birth was reported in 40 patients (12.7%). In 240 patients whose age at diagnosis was specified, the distribution presented a short peak at < 1 year old with a long tail in older age (Additional file 3).

**Table 1:** Patient characteristics (the past 30 years; 1985–2015)

	Total (*n* = 316) n (%)	Age at diagnosis < 18 (*n* = 92) n (%)	Age at diagnosis ≥18 (*n* = 148) n (%)	*P* value (chi-square test)
Gender				
Male	171 (54.1)	43 (46.7)	87 (58.8)	0.069
Female	145 (45.9)	49 (53.3)	61 (41.2)	
Age at diagnosis				
< 1 year old	24 (7.6)	24 (26.1)	–	–
1–17 years old	68 (21.5)	68 (73.9)	–	–
≥ 18 years old	148 (46.8)	–	148 (100.0)	–
NA^a^	76 (24.1)	–	–	–
Onset of respiratory symptoms				
Neonatal or infant	60 (19.0)	47 (51.1)	9 (6.1)	1.10E-15
Childhood or adolescent	131 (41.5)	35 (38.0)	74 (50.0)	0.070
Adult	28 (8.9)	–	21 (14.2)	–
NA	97 (30.7)	10 (10.9)	44 (29.7)	6.69E-04
Reason for medical consultation				
Cough	90 (28.5)	31 (33.7)	56 (37.8)	0.516
Dyspnea	64 (20.3)	28 (30.4)	33 (22.3)	0.159
Fever	28 (8.9)	10 (10.9)	18 (12.2)	0.762
Infertility as complaint^a^				
Male	24 (19.2)	–	–	–
Female	1 (1.1)	–	–	–
Hemoptysis	18 (5.7)	3 (3.3)	12 (8.1)	0.132
Preoperative examination for other disease	21 (6.6)	6 (6.5)	7 (4.7)	0.551
Chest x ray checkup without symptoms	8 (2.5)	4 (4.3)	4 (2.7)	0.490
Medical history				
Bronchial asthma	19 (6.0)	10 (10.9)	9 (6.1)	0.182
Diffuse panbronchiolitis	8 (2.5)	0	5 (3.4)	0.075
Pulmonary tuberculosis	8 (2.5)	0	8 (5.4)	0.023
Pulmonary nontuberculous mycobacterial infection	3 (0.9)	0	1 (0.7)	0.430
Recurrent pneumonia	99 (31.3)	34 (37.0)	51 (34.5)	0.694
Otitis media	62 (19.6)	17 (18.5)	27 (18.2)	0.964
Congenital heart disease	8 (2.5)	8 (8.7)	0	2.64E-04
Rhinosinusitis	246 (77.8)	53 (57.6)	127 (85.8)	9.31E-07
Bronchiectasis	221 (69.9)	47 (51.1)	116 (78.4)	1.06E-05
Chronic bronchitis	8 (2.5)	4 (4.3)	2 (1.4)	0.148
Situs inversus	200 (63.3)	50 (54.3)	98 (66.2)	0.066
Family history				
PCD family history	26 (8.2)	8 (8.7)	14 (9.5)	0.842
Consanguineous parents	15 (4.7)	2 (2.2)	12 (8.1)	0.057
Reproductive history^b^				
Spontaneous conception				
Male	8 (14.3)	–	–	–
Female	11 (44.0)	–	–	–
Infertility as history				
Male	48 (85.7)	–	–	–
Female	14 (56.0)	–	–	–

*NA* (not available)

^a^When a patient was suspected of having PCD and diagnosed, a doctor in charge published the case report, describing the patient’s age when the diagnosis was made. When this description was not found in the report, it was categorized as NA

^b^Information of infertility was available from 125 male and 94 female patients ≥18 at the visit to clinician. Reproductive history was available in 56 male and 25 female patients

Patient characteristics were further compared between two groups with age at diagnosis < 18 years and ≥ 18 years in Table 1. Medical history of congenital heart disease (8 [8.7%] vs. 0 patients) showed significantly higher frequency in the younger group (*P* value = 2.64E-04). Description of previous pulmonary tuberculosis (0 vs. 8 patients [5.4%]; *P* value = 0.023), rhinosinusitis (53 [57.6%] vs. 127 patients [85.8%]; *P* value = 9.31E-07), and bronchiectasis (47 [51.1%] vs. 116 patients [78.4%]; *P* value = 1.06E-05) showed significantly higher frequency in the older group. In medical and family history, however, it is difficult to distinguish unawareness from absence of diseases. For instance, bronchiectasis is present in 47 (51.1%), absent 4 (4.3%), and not described in 41 (44.6%) under 18 years.

Of the patients whose reproductive history was available, 8 (14.3%) of 56 male patients had spontaneously fathered children, and the rest, 48 (85.7%) had no description about children and possibly infertile (Table 1); 15 of which received assisted reproductive technologies (ART) and 12 obtained children. Of 25 female patients, 11 (44.0%) became pregnant spontaneously and 14 (56.0%) were possibly infertile, though the details were not described. One delivered a child after ART.

### Laboratory findings

Laboratory findings were summarized and further compared between two groups of patients whose age at diagnosis < 18 years and ≥ 18 years in Table 2. Age at visit to clinical institutes was 7.5 years (1–13) in those < 18 years and 37.5 years (29–59) in those ≥18 years (*P* value = 2.20E-16). Respiratory function tests showed mild decrease in vital capacity (VC) in both groups (VC [% predicted]; 68.3% [±30.7] vs. 71.2% [±21.4]). Airflow obstruction was more severe in patients ≥18 than < 18 (forced expiratory volume in one second/forced vital capacity [FEV_1_/FVC] ratio [%]; 75.7% [±12.7] vs. 60.3% [±16.1]) (*P* value = 0.005). A significant negative correlation was observed between their FEV_1_/FVC ratio (%) and their age at the visit to clinicians as well as age at diagnosis (Spearman’s rank correlation coefficient *rho* = − 0.293 and *P* value = 0.010; *rho* = − 0.278 and *P* value = 0.046, respectively).

**Table 2:** Laboratory findings (the past 30 years; 1985–2015)

A. measurements of respiratory function tests				
	Total (*n* = 316) Mean (±SD)	Age at diagnosis < 18 (*n* = 92) Mean (±SD)	Age at diagnosis ≥18 (*n* = 148) Mean (±SD)	*P* value^a^
VC (% predicted)	*n* = 63	*n* = 10	*n* = 37	
	71.3 (±22.5)	68.3 (±30.7)	71.2 (±21.4)	0.650
FEV_1_/FVC ratio (%)	*n* = 77	*n* = 10	*n* = 42	
	63.3 (±15.0)	75.7 (±12.7)	60.3 (±16.1)	0.005
FEV_1_ (% predicted)	*n* = 9	*n* = 3	*n* = 6	
	48.1 (±27.4)	35.4 (±29.7)	54.4 (±26.5)	0.262
RV/TLC ratio (%)	*n* = 26	*n* = 1	*n* = 8	
	44.2 (±15.1)	24.3	45.5 (±17.3)	0.444
B. frequencies of pathogens detected in bacterial cultures and frequencies of other tests				
	Total (*n* = 316) n (%)	Age at diagnosis < 18 (*n* = 92) n (%)	Age at diagnosis ≥18 (*n* = 148) n (%)	*P* value (chi-square test)
Bacterial sputum culture	*n* = 75	*n* = 10	*n* = 41	
*Pseudomonas aeruginosa*	32 (42.7)	5 (50.0)	13 (31.7)	0.278
*Haemophilus influenzae*	27 (36.0)	2 (20.0)	15 (10.1)	0.319
*Klebsiella pneumoniae*	7 (9.3)	0	4 (9.8)	0.304
*Streptococcus pneumoniae*	6 (8.0)	2 (20.0)	3 (7.3)	0.227
*Staphylococcus aureus*	2 (2.7)	0	2 (4.9)	0.476
*Aspergillus fumigatus*	2 (2.7)	0	2 (4.9)	0.476
Normal flora	12 (16.0)	2 (20.0)	8 (19.5)	0.972
Sputum acid-fast bacteria culture	*n* = 17	*n* = 2	*n* = 14	
*Mycobacterium avium* complex	5 (29.4)	0	4 (28.6)	0.383
*Mycobacterium tuberculosis*	1 (5.9)	0	1 (7.1)	0.696
Negative	11 (64.7)	2 (100)	9 (64.3)	0.308
Saccharin test	*n* = 33	*n* = 8	*n* = 25	0.073
Nasal NO test	*n* = 4	*n* = 2	*n* = 2	0.628

VC (vital capacity), FEV_1_ (forced expiratory volume in one second), FVC (forced vital capacity), RV (residual volume), TLC (total lung capacity), Nasal NO (nasal nitric oxide)

^a^Exact Wilcoxon rank sum test

Sputum culture results were available in 75 patients. *Pseudomonas aeruginosa* (*P. aeruginosa*) (age at diagnosis < 18; 5 patients [50.0%], age at diagnosis ≥18; 13 patients [31.7%]) was most frequently detected in both groups. Median age at diagnosis of patients with *P. aeruginosa* infection was 36 years (IQR 17.25–45.5). Sputum acid-fast bacteria culture was tested in 17 patients. *Mycobacterium avium* complex (4 patients [28.6%]) and *Mycobacterium tuberculosis* (1 patient [7.1%]) were detected only in the older group. Median age of the patients with *Mycobacterium avium* complex infection was 54 years (IQR 28–79.25).

### EM findings

EM analysis of respiratory epithelial cells and sperm for ultrastructural examination of axonemes had been performed in 250 specimens from 230 patients (72.8%). Of these, 210 patients were assessed with one specimen type (210 specimens), and 20 patients (8 patients with bronchial and nasal mucosa, 4 patients with bronchial mucosa and sperm, and 8 patients with nasal mucosa and sperm) with two different specimen types (40 specimens).

Data of 226 specimens, excluding 24 specimens with inconsistent findings were summarized (Table 3). Bronchial mucosa (119 specimens [52.7%]) was more frequently assessed than nasal mucosa (90 specimens [39.8%]) and sperm (17 specimens [7.5%]). The most frequent ultrastructural abnormalities were defects of dynein arms. Both outer dynein arm (ODA) and inner dynein arm (IDA) defects, IDA defects and ODA defects were seen in 57 specimens (25.2%), 45 (19.9%) and 14 (6.2%), respectively. IDA defect may include IDA defect with microtubule disorganization (MTD), not widely recognized until causative genes were identified in 2013 [18]. When IDA-defect based and both ODA and IDA-defect based abnormalities were subtotaled respectively, they were seen in 56 specimens (24.8%) and 63 specimens (27.9%), respectively. Both MTD and central apparatus (CA) defects without dynein arm defects were seen in 3 specimens (1.3%). No structural abnormalities were detected in 4 specimens (1.8%). Simply, “dynein arm defect” and “abnormal cilia” were described in 34 specimens (15.0%) and 6 specimens (2.7%), respectively. Radial spokes defects were described in 3 specimens, but omitted from this table because of difficulties in proper assessment [11, 19].

**Table 3:** Electron microscopy findings (the past 30 years; 1985–2015)

EM findings	Site of biopsy n (%)			Total (*n* = 226)^a^
	Nasal mucosa (*n* = 90)	Bronchial mucosa (*n* = 119)	Sperm (*n* = 17)	
No structural abnormalities	1 (1.1)	2 (1.7)	1 (5.9)	4 (1.8)
IDA defects^b^	21 (23.3)	24 (20.2)	0	45 (19.9)
IDA defects and MTD^b^	5 (5.6)	1 (0.8)	1 (5.9)	7 (3.1)
IDA defects and CA defects	0	2 (1.7)	0	2 (0.9)
IDA defects, CA defects and MTD	1 (1.1)	1 (0.8)	0	2 (0.9)
ODA defects	6 (6.7)	8 (6.7)	0	14 (6.2)
Both ODA and IDA defects	23 (25.6)	28 (23.5)	6 (35.3)	57 (25.2)
Both ODA and IDA defects and MTD	2 (2.2)	1 (0.8)	2 (11.8)	5 (2.2)
Both ODA and IDA defects and CA defects	0	1 (0.8)	0	1 (0.4)
Only MTD	1 (1.1)	0	2 (11.8)	3 (1.3)
Only CA defects	2 (2.2)	0	1 (5.9)	3 (1.3)
MTD and CA defects	0	2 (1.7)	0	2 (0.9)
Complicating compound cilia	6 (6.7)	11 (9.2)	0	17 (7.5)
Abnormal cilia^c^	2 (2.2)	4 (3.4)	0	6 (2.7)
Dynein arm defect^c^	8 (8.9)	22 (18.5)	4 (23.5)	34 (15.0)
NA	12 (13.3)	12 (10.1)	0	24 (10.6)

IDA (inner dynein arm), ODA (outer dynein arm), MTD (microtubule disorganization), CA (central apparatus), NA (not available)

^a^EM findings from 24 specimens (12 patients) were inconsistent between two specimen types, and excluded from this table

^b^“IDA defects” may include IDA defects with MTD not reported during the study period

^c^Abnormal cilia or dynein arm defects with no further detailed description

We summarized EM findings in 6 groups; IDA defects (*n* = 55: 23.9%), ODA defects (14; 6.1%), both ODA and IDA defects (57; 24.8%), other structural abnormalities (25; 10.9%), no abnormalities (4; 1.7%), and no definite conclusion or description (75; 32.6%) (Additional file 4). We also listed EM findings from the same specimen types (vs. Boaretto et al.; total 215 patients whose EM findings found from nasal or bronchial mucosae, vs. Kennedy et al., Noone et al. and de Iongh et al.; total 99 patients whose EM findings found from nasal mucosae). In their data, ODA defect and both ODA and IDA defect were predominant (Additional file 4).

### Other diagnostic tests

Nasal NO measurement was performed in 4 patients (1.3%) in total. All the patients were reported after 2012. The saccharin test was performed in 33 patients (10.4%) until 2014. During the search period, 1985 to 2015, mutation screening of two PCD-causing genes, *DNAH5* and *DNAI1*, was performed in four patients, of which a sibling pair carried the same homozygous variant in *DNAI1* (NM_012144.3: c.1163G > A, p. C388Y), which was subsequently demonstrated as a disease-causing mutation in a recent study [20]. IF test and HSVM were not performed in any of our patients.

### Treatment and outcome

Although not shown in the table, long-term macrolide therapy was most commonly used (erythromycin; 37 patients 11.7%, clarithromycin; 21 patients 6.6% and azithromycin; 3 patients 0.9%) followed by respiratory physiotherapy (25 patients 7.9%), surgical therapy (23 patients 7.3%). Among patients receiving surgical therapies, lobectomy (9 patients 2.8%) and pneumonectomy (2 patients 0.6%) were performed due to hemoptysis or lung abscess. Lung transplant was performed in 2 patients (0.6%) with severe respiratory failure. Among 106 patients (33.5%) whose changes in respiratory or general conditions were described, their signs and symptoms were once improved in 85 patients (26.9%), worsened in 5 patients (1.6%) and resulted in death in 16 patients (5.1%). All these 16 patients died within 5 years of diagnosis, and their median age at death was 41 years (IQR 23–57.8). Onset of their respiratory symptoms was during neonatal or infant period in 3 patients, childhood or adolescent period in 8 patients, and adulthood in 5 patients. Only 2 patients died of heart disease in neonatal or infant period, 11 patients died of respiratory infection, and 3 patients died of malignant tumor; 2 lung cancers and 1 malignant lymphoma. The duration from time of diagnosis or treatment to death was variable, and there were no significant associations between outcomes and parameters including age at diagnosis, age at onset of respiratory symptoms and treatment measures. (data not shown).

### Subgroup analysis stratified by EM findings

Results of EM tests were classified into four groups; IDA defects, ODA defects, both ODA and IDA defects, and others, but none of these defects showed any significant associations with clinical characteristics including Kartagener triad and infertility (data not shown).

### Risk of bias analysis

In this review, 197 patients (62.3%) were reported in original articles and 119 patients (37.7%) were reported in only conference abstracts. Infertility (51 vs. 11 patients), bronchiectasis (152 vs. 69 patients), rhinosinusitis (169 vs. 77 patients), otitis media (48 vs. 14 patients) and bacterial sputum test results (71 vs. 4 patients) were less frequently reported in conference abstracts than in original articles (*P* values = 3.06E-04, 3.16E-04, 1.23E-05, 0.006 and 3.70E-11, respectively). Frequencies of major EM findings were not significantly different between the articles published in 1985 to 2000 and 2001 to 2015 (data not shown).

## Discussion

This is the first systematic review and meta-analysis containing more than 300 Japanese patients with PCD, before modern diagnostic tools such as extensive genetic testing were started to use. Kartagener triad was observed in more than 60% of cases in this review. A majority of patients survived over 18 years, mostly suffering from chronic respiratory infection, and the diagnosis of PCD was not established often until adulthood. During the study period, the past 30 years, observation under EM was a main diagnostic method for PCD, and therein IDA defects rather than ODA defects were often reported.

Prevalence of PCD in Japanese is estimated as one in 8000 to 10000 on the assumption that approximately a quarter of individuals with situs inversus (one in 4000 to 5000 in Japan [13]) have PCD and that almost a half of PCD patients have situs inversus [10, 21]. This proportion may be larger than those in western countries, indicating that many patients remain unrecognized [14, 22].

Typical PCD patients are known to present respiratory symptoms in early infancy. Situs inversus is observed in almost half of patients, and nearly all develop bronchiectasis by their adulthood [10, 16, 23, 24]. We should consider several reasons why nearly half of the patients’ age at diagnosis were ≥ 18 years, why less than 80% of the adult patients described bronchiectasis, why almost 70% of the adult patients showed situs inversus, and why almost 10% started to show respiratory symptoms even after 18 years old in our study. First, the diagnosis is delayed because of underestimation and unawareness of the disease, and of poor accessibility to diagnostic tests in the pediatric field. A small percentage of case reports from pediatricians in our study support this idea. Second, making a definite diagnosis itself is challenging during the time when EM was the only specific test that clinicians rely upon. Patients diagnosed as having PCD with no ultrastructural abnormalities were very few in our study, although currently experts believe that approximately 30% of patients with PCD have normal or nearly normal [25]. Third, PCD is a genetically heterogeneous disorder and some of the adult patients in our study may have milder phenotype than those reported previously. We have recently identified an adult patient with a unique large deletion variant of *DRC1* gene that causes PCD with normal or nearly normal EM findings, presumably common to Asians, but uncommon to non-Asians [26]. Accumulation of such patients and their geographic origin may provide us more insight into Asian PCD. Compared to a systematic review by Goutaki et al. from Switzerland, we also showed the low frequencies of otitis media (age at diagnosis < 18 18.5% and age at diagnosis ≥18 18.2% vs. weighted mean 73%, range 23–100%) and neonatal respiratory distress at full term birth (12.7% vs. weighted mean 51%, range 15–91%) [6].

Although there are no specific treatment measures at present, and our review could not fully assess the treatment effects, it is likely that the prognosis is improved when the patients are diagnosed early and their complications are managed properly [27, 28]. Early diagnosis also enables patients and their families to receive chances of appropriate genetic counseling, and screening for PCD-associated ear, rhino-sinus, and pulmonary diseases with infertility [29].

Similar to previous reports, chronic pulmonary infection caused by nontuberculous mycobacteria and *P. aeruginosa*, and male infertility may be the first clue to suspect PCD, when other symptoms and clinical phenotype are not recognized until adulthood [10, 16, 30, 31]. This review showed that diffuse panbronchiolitis (DPB) was reported as a previous illness in 8 PCD patients. DPB is a chronic respiratory disease whose signs and symptoms are quite similar to PCD and prevalent in East Asian countries, whereas cystic fibrosis (CF) is quite rare in Asia [32–34]. Undiagnosed PCD may be hidden among such a disorder [26], and should be carefully assessed [35, 36].

Nasal NO measurement is recommended as a useful test to support the diagnosis of PCD. However, it has rarely been performed in Japan [3, 7, 37]. According to the recent reports, CF and DPB also show relatively low nasal NO [38, 39]. Nevertheless, a nasal NO cutoff value < 77 nl/min may be suitable to detect PCD, because this cut off value demonstrated 98% of sensitivity and > 99% of specificity for the patients diagnosed as having PCD with ciliary axonemal defects or mutations in *DNAH11* [37]. After careful determination of the cutoff value and standardization of the method in Japan, nasal NO measurement along with the increased awareness of PCD may be an initial step of screening before performing expensive genetic tests.

In general, many PCD causative genes are related to ODA defects or to both ODA and IDA defects, and only two (*CCDC39* and *CCDC40*) have been reported to cause IDA defects with MTD [18, 40]. It is well known that structural defects of IDA were historically a major disturbance in making a correct diagnosis. Many reports have shown that IDA defects without other abnormalities could result from artifacts or secondary changes of cilia in the inflammatory background [29, 41], and PCD that originally retains normal ciliary ultrastructure may also show apparent IDA absence. Studies on flagella of *chlamydomonas* and other organisms identified several genes related to organization of IDA. However, it remains unknown whether PCD with IDA defects alone could be found as genetic defects of such human orthologs [42–44].

Considering all, IDA defects frequently reported are possibly caused by 1) variants of known PCD genes that cause IDA defects accompanied by MTD or other abnormalities overlooked (ex. *CCDC39* and *CCDC40*); 2) pathogenic PCD gene variants that show normal ciliary structure (ex. *DNAH11*) with secondary IDA absence; 3) variants of unidentified PCD genes that cause primary IDA defects unique to Japanese; or 4) other chronic respiratory diseases combined with secondary IDA absence. Because 10 patients with IDA defects presented the classical Kartagener triad in our review, a certain type of PCD should be present at least in these patients.

This systematic review has several limitations. First, frequencies of their signs and symptoms may be underestimated, particularly because adult patients do not recall their respiratory symptoms or medical history in the early days, and because clinical information other than point of focus in their specialized reports tended to be insufficient, and information in conference abstracts was generally more often lacking than in original articles. Second, EM analytical methods and other diagnostic and treatment procedures were not standardized in PCD; the experience of diagnostic scientists in Japanese test centers is likely to be hugely variable, and treatment period and type of therapy were not objectively assessed. Third, overestimation of EM findings using a single specimen together with publication bias may increase the false-positive results. Diagnostic methods of PCD have been evolving rapidly, and awareness of the disease particularly in the pediatric field may totally change the entire clinical picture of Asian PCD in the near future. Patients diagnosed within one year should be increased and those over 18 years with delayed diagnosis should be decreased.

## Conclusion

In this report, we systematically reviewed the situations and problems of PCD experienced for the past 30 years in Japan. The diagnosis of PCD was often delayed and challenging. Because of ethnic differences and insufficient resources during the study period, we should consider several possibilities to interpret EM findings in Japanese patients. Establishment of accurate diagnostic pipelines including appropriate genetic and other modern testing methods, and better management system are urgently needed.

## Additional files

Additional file 1: The details of the search strategy (MEDLINE, EMBASE, and Japana Centra Revuo Medicina in Japanese). (XLSX 12 kb)

Additional file 2: PRISMA checklist. (DOCX 30 kb)

Additional file 3: Distribution of age at diagnosis (*n*=240). (TIF 403 kb)

Additional file 4: Summary of EM findings in the present study with other international literature data. (XLSX 46 kb)

## Data Availability

The datasets used and/or analysed during the current study are available from the corresponding author on reasonable request.

## Abbreviations

ART: Assisted reproductive technologies
CA: Central apparatus
CF: Cystic fibrosis
DPB: Diffuse panbronchiolitis
EM: Electron microscope
FEV_1_: Forced expiratory volume in one second
FVC: Forced vital capacity
HSVM: High speed videomicroscopy
IDA: Inner dynein arm
IF: Immunofluorescence
MTD: Microtubule disorganization
NA: Not available
NO: Nitric oxide
ODA: Outer dynein arm
PCD: Primary ciliary dyskinesia
PRISMA: Preferred Reporting Items for Systematic Reviews and Meta-Analyses
RV: Residual volume
TLC: Total lung capacity
