# Supplementary figures and images for: Primary ciliary dyskinesia in Japan: systematic review and meta-analysis
Source: BMC Pulm Med. 2019 Jul 25;19:135. doi: 10.1186/s12890-019-0897-4 (PMC6659197; doi:10.1186/s12890-019-0897-4)

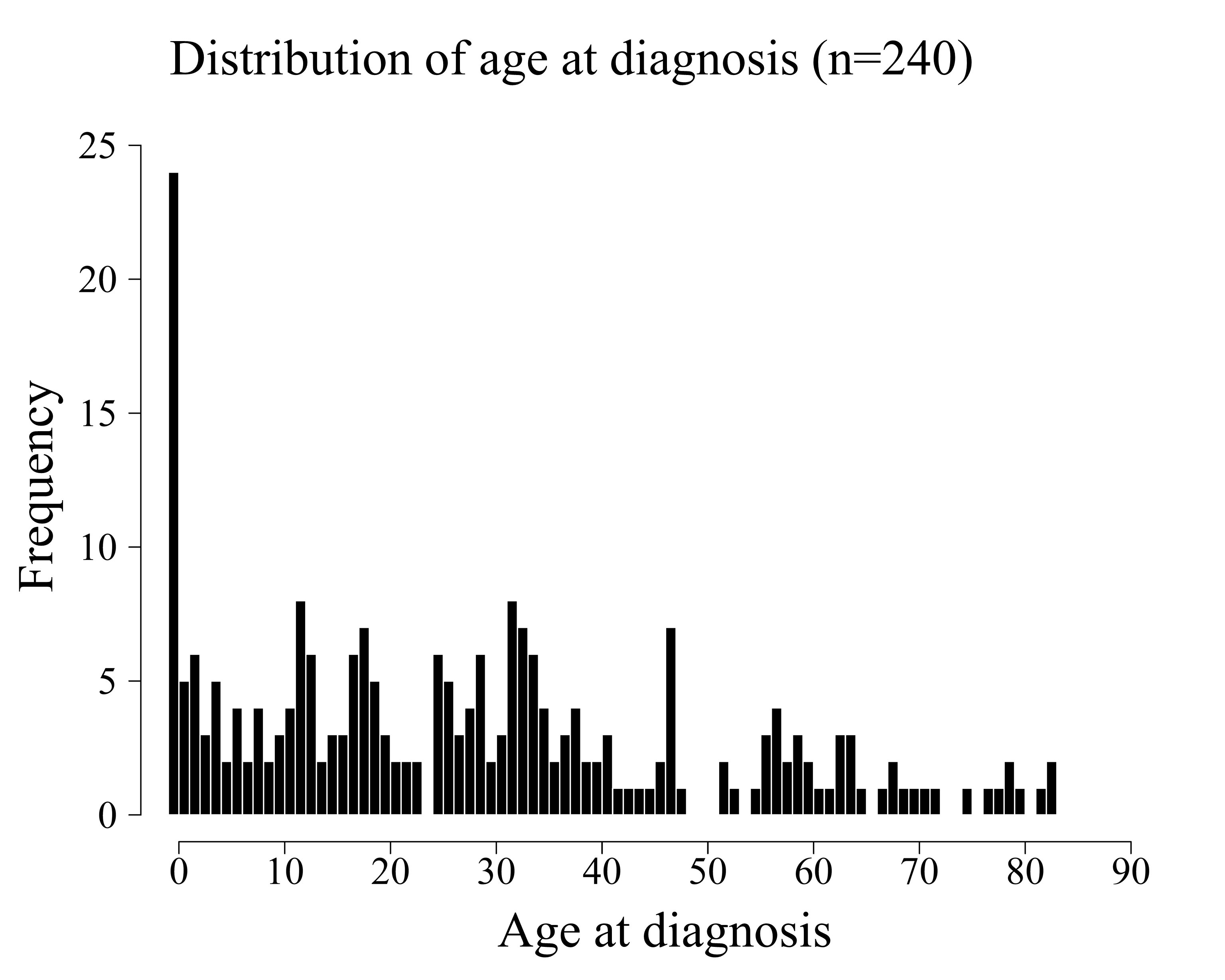

Supplement: Supplementary file 3 — Distribution of age at diagnosis (n=240). (TIF 403 kb) [file 12890_2019_897_MOESM3_ESM.tif]
